# Structural Power and Epistemologies in the Scientific Field: Why a Rapid Reconciliation Between Functional and Evolutionary Biology is Unlikely

**DOI:** 10.1007/s11024-023-09520-0

**Published:** 2024-01-30

**Authors:** Pierre Benz, Felix Bühlmann

**Affiliations:** 1 Faculty of Social and Political Sciences, Institute of Political Studies, University of Lausanne, Lausanne, Switzerland; 2 Faculté des arts et des sciences, École de bibliothéconomie et des sciences de l’information, Université de Montréal, Montréal, Canada; 3 Faculty of Social and Political Sciences, Institute of Social Sciences, University of Lausanne, Lausanne, Switzerland

**Keywords:** Evolutionary biology, Functional biology, Disciplines, Scientific field, Epistemologies

## Abstract

The past decade has been marked by a series of global crises, presenting an opportunity to reevaluate the relationship between science and politics. The biological sciences are instrumental in understanding natural phenomena and informing policy decisions. However, scholars argue that current scientific expertise often fails to account for entire populations and long-term impacts, hindering efforts to address issues such as biodiversity loss, global warming, and pandemics. This article explores the structural challenges of integrating an evolutionary perspective, historically opposed to functional determinants of health and disease, into current biological science practices. Using data on Swiss biology professors from 1957, 1980, and 2000, we examine the structural power dynamics that have led to the division between these competing epistemologies, and how this division has influenced resource allocation and career trajectories. Our analysis suggests that this cleavage presents a significant obstacle to achieving fruitful reconciliations, and that increased academicization and internationalization may benefit functional biologists at the expense of evolutionary biologists. While evolutionary biologists have gained symbolic recognition in recent years, this has not translated into valuable expertise in the political domain.

## Introduction

In recent times, the world has been confronted with a series of major crises, such as global warming, biodiversity crisis, and the emergence of the Covid-19 pandemic since 2020. Concomitantly, scholars in the fields of the sociological and biological sciences have emphasized the need for more integration of their expertise in the making of public policies (Saint-Martin 2020; Alizon 2020; Gaudillière et al. 2021). Major crises have indeed the potential to revolutionize our thinking about the world and the relationship between science and politics. However, such a call for inter- but also intra-disciplinary conciliation is not new. Especially biologists who later took on the role of historians of their discipline have been calling for a for a more integrated science of life, while decrying the persisting lack of cooperation between biologists. These ‘progressive’ biologists emphasize the difficulty of transgressing the barriers set up by ‘conservative’ biologists who are accused of not being supportive of epistemological reconciliation (Laland et al. 2011; Morange 2011).

The field of biological sciences is diverse, ranging from micro-level biology aimed at developing (for instance) vaccines to the study of evolutionary and ecological issues. Although the complementarity between the two for addressing major issues such as pandemics has been assessed over the past decades (Lederberg 1988; O’Malley 2009; Alizon and Méthot 2018), today’s call upon the biological sciences to guide political decisions has not served as a catalyzer for a meeting of perspectives. Instead, bioscientists have been integrated to various degrees into taskforces and the granting of funding, with evolutionary approaches suffering from a consistent lack of consideration by the public authorities. An evolutionary approach is however essential because it focuses on long-term dynamics, including viruses and microbial populations. For instance, when deciding on the use of antibiotics or vaccines in the face of a large-scale infection, it is vital to balance short-term benefits against the evolution of resistances, which could potentially result in a loss of effectiveness (Alizon 2020). The population-level phenomena raised by evolutionary biologists are therefore critical to implementing public policies aimed at fighting and anticipating crises. Hence, the reason for the evolutionary biologists’ lack of symbolic legitimacy is not a decontextualized scientific issue, but linked to structural, political and historical differentiations that endow them with fewer credibility.

This article seeks to examine the power dynamics within the biological sciences between 1957 and 2000 and how they relate to the symbolic value historically attributed to functional and evolutionary epistemologies. Symbolic hierarchies refer to the perceived value attached to different and often competitive ways of framing research questions and conducting research (Bourdieu 1991; Gingras 2012; Lamy and Saint-Martin 2018). In this regard, we aim to address the following questions: How has the credibility of different sub-disciplines of the biological sciences changed over time? How are scientific, extra-scientific, and international resources allocated across these sub-disciplines? And how do these differences reflect a divide between evolutionary and functional epistemologies? To answer these questions, we analyze the careers of all tenured (full and associate) professors of biological sciences in Switzerland in 1957, 1980, and 2000 (n=442) by using multiple correspondence analysis (MCA). This method reduces the variance of an entire population’s resource endowment into principal explanatory dimensions (Le Roux and Rouanet 2010; Hjellbrekke 2018). We explore the changing distribution of valuable scientific, extra-scientific, and international resources among professors, while also distinguishing between institutional disciplinary affiliations and epistemologies. Professors are key actors in the production and transmission of knowledge. Therefore their training and research practices are crucial to understand the power dynamics underlying the formation and maintenance of knowledge and disciplines (Bourdieu 2001; Morgan et al. 2022; Rossier and Benz 2022). The Swiss case is particularly relevant due to its significant contribution to the development of molecular biology, alongside Germany and the United States (Strasser 2006). Moreover, its highly competitive higher education system provides an ideal context to study the hierarchies between two competing epistemologies (Stettler 2002; Benninghoff and Braun 2010).

Our findings reveal a persistent divide between functional and evolutionary biologists throughout the latter half of the 20^th^ century. This division is sustained by a differentiated allocation of scientific, extra-scientific, and international resources among professors. Our analysis suggests that scientific credibility is closely linked to power structures, namely the distribution of material and symbolic resources among professors. We contend that epistemologies reflect specific interests that result from struggles within the scientific field and the resulting scientific positions occupied by scholars (Bourdieu 1984). Epistemologies do not inherently possess the capacity to transform internal contradictions into new logics, as demonstrated by Hegel’s *Selbstbewegung*. Instead, their potential for transformation depends on the space of possibilities offered by political contexts and ‘material’ historical conditions of scientific activity (Bourdieu 1991, 1992; Rheinberger 2010; Meloni 2016). We emphasize the importance of the unequal distribution of resources and the highly competitive nature of scientific activity. Therefore, we do not anticipate a swift reconciliation of epistemologies in the biological sciences. Instead, our contribution underscores the powerful inertia of institutional disciplinary structures in shaping scientific hierarchies, careers, and rewards, raising doubts about biology’s capacity to offer (political) responses to the major crises of our time.

The structure of this article is as follows: First, we present our conceptual framework. Second, we describe our research strategy, data sources, and methodology. Third, we demonstrate how both evolutionary and functional epistemologies are rooted in academic power relations and linked to professors’ resource endowments. Following our results, we conclude by discussing the contributions of this article to the literature and calling for additional research on the relationship between epistemologies and academic power.

### Diverging Epistemologies as a Historical and Social Process

While traditional evolutionary and molecular biology have been seen as separate epistemological domains, recent contributions in biology propose strategies to bridge the gap between the two by pointing to possible convergences in the use of common terms such as organism, interactionism, adaptation, or gene (Barberousse et al. 2009; Morange 2011; Laland et al. 2011). However, others remain skeptical and emphasize the lack of social and institutional conditions necessary for a widespread integration of epistemologies (Keller 2014; Larregue et al. 2020). These barriers were explicitly problematized at the beginning of the second half of the twentieth century. The most striking example is Mayr’s seminal article (1961), in which he argued that a deep epistemological divide exists between two concurrent accounts in the biological sciences regarding life and its causes. Functional biologists, asking *how*, focus on proximal causes and propose direct relationships between cause and effect at the microbiological level. Evolutionary biologists, asking *why*, focus on historical explanation of ultimate causes at the macro level. These epistemic differences are fundamental, right down to the way the two groups define themselves. Comparing the scientific work of Swiss biologists, Stettler (2002, 198–199) shows, for example, that functional biologists often described their activity as ‘choosing the simplest procedure’, ‘making great progress’, aiming at understanding ‘fundamental laws of nature’, and ‘educating the elite’. Evolutionary biologists mentioned preferably ‘producing a lot of expensive work’, ‘not jumping to conclusions’, ‘giving insight into diversity and a better understanding of complex systems’, and the need for ‘training local scientists’.

Mayr’s critique was timely, coinciding with a pivotal moment in biology. In 1953, Watson and Crick’s characterization of DNA’s double helix structure not only revolutionized genetics but also imbued biology with a renewed commitment to ‘reductionism’ (Wilson 1994). What Wilson named after the ‘molecular war’ in turn led to a need for ‘traditional’ biologists to engage in a process of redefining themselves, eventually through the label of ‘evolutionary biology’. Consequently, institutions supporting scientific endeavors, such as the Swiss National Science Foundation in the Swiss case, embraced this distinction, eventually classifying them as ‘biology I’ for evolutionary biology and ‘biology II’ for functional biology. During this period, both traditional and emerging biologists grappled with the central question of genes, albeit through divergent approaches. The post-World War II era witnessed a growing interest in the ‘modern synthesis’ among evolutionary biologists, with Mayr as a notable advocate. This framework viewed evolutionary mechanisms as dynamic processes spanning genes, organisms, and populations, albeit distinct from the formal genetics it originated from (Morange 2020: 23). Conversely, what would later become molecular biology was not an institutionalized discipline, but an ‘assemblage’ of diverse disciplines and corresponding instruments, which often had ‘nothing to do’ with biology (Rheinberger 2010).

This era marked the beginning of what François Gros (2003), former director of the Institut Pasteur, depicts as a ‘half-century of transformations’ of the biological sciences. From this period onwards, the symbolic hierarchy between functional and evolutionary biology has reinforced, with functional biology continuing to receive strong material and financial support. As in the US, the Swiss molecular biologists have received massive public funding since the creation of the Swiss National Science Foundation in the early 1950s. These fundings even increased from the mid-1960s onwards. The new science policy saw the potential of the ‘discovery of the genetic code’ as ‘the greatest step ever taken towards understanding the continuity of life’ and consequently advantaged functional biology (Stettler 2002: 158).

The period of institutionalization, from 1965 to 1972 saw an increase in the number of groups and laboratories working with molecular biology, with an expansion of the molecular vision beyond its original field, leading to a takeover of other biological disciplines by molecular scientists. In addition to important funding, often at the expense of other researchers, molecular biologists took control over scientific journals and in the universities, either by introducing molecular biology in the curricula or, more often, by updating biochemistry or genetics courses (Morange 2020: 171). The second ‘assemblage’ of molecular biology through genetic engineering has further accentuated the loss of symbolic legitimacy for evolutionary biology (Reinhardt 2002; Chandler 2005; Bürgi 2005; Strasser 2006; Stettler 2002). The biotech revolution has only deepened this divide, with gene becoming the equivalent of the atom for biology and leading to the emergence of big science in biology (Keller 2005; Gingras 2012; Benz 2022).

### A Structural Take on the Relationships of Power in the Swiss Biological Sciences

Epistemologies are embodied in different ways in scientific disciplines and are subject to historical variations (Abbott 2001). Meloni (2016) introduced the notion of ‘political biology’ as an application of Shapin and Schaffer’s (1984) ‘political epistemology’ to the history of biology. Moreover, epistemologies settle at the interface of scientific statement and political order. The sense of notions and concepts are to be understood with knowledge of the context in which they were produced. Thus, one must consider epistemologies as culturally, socially, and politically grounded, and resulting from the specific contexts from which they are generated and disputed (Jasanoff 2005). These are processes and the history of biology has not fostered the understanding of life around a few key notions, such as genetic determinism; rather, it has brought critical challenges to such notions (Keller 2000). Therefore, we understand epistemology not as the analysis aiming at clarifying what constitutes knowledge, but as ‘the conditions under which and the means with which things are made into objects of knowledge’ (Meloni 2016: 224; Rheinberger 2010). This definition of epistemology as historical and political process goes not without reminding of Marx’s historical materialism (Gingras 2010). It also directly echoes Bourdieu’s research program that identifies epistemological conflicts as always, inseparably, political conflicts (Bourdieu 1976).

To account for these processes, we aim to investigate the connection between biological epistemologies and the changing institutional conditions of scientific research. Indeed, we assume that power relations that determine the structure of the relationship between functional and evolutionary biologists have progressively become institutionalized. Furthermore, both groups are dependent on the same environment when it comes to competing for scientific credibility (Parker et al. 2010). The scientific community functions as a social ecosystem that produces scientific credibility through specific mechanisms, which leads to significant disparities among scholars in terms of career progression, scientific prizes, and citations, with a minority of scholars possessing most of the resources (Merton 1968; Larivière et al. 2010; Bol et al. 2018). Using the concept of field, we can analyze the link between the discipline's status and the collective volume and type of legitimate resources held by influential scholars (Bourdieu 2001). The hierarchy of disciplines encompasses scientific credibility resources, institutional power, and extra-academic experiences in a context of increasing internationalization of knowledge production and academic trajectories (Bourdieu 1984; Braun 2001; Gingras 2002). We differentiate between scientific, extra-scientific, and international resources. Scientific resources relate to the accumulation of symbolic credibility and knowledge recognition by peers (Bourdieu 1976). Extra-scientific resources include leadership positions in scientific organizations or universities, executive power, and experiences gained through careers in the public or private sector (Bourdieu 1997; Benninghoff and Braun 2010). International resources relate to the symbolic value associated with experience gained abroad (Bühlmann 2020).

Despite significant historical changes, the social system of science has not undergone radical transformation. The symbolic hierarchy of disciplines remains relatively stable, with only limited susceptibility to change, according to Bourdieu and Wacquant (1992). Academia is defined as a field by Bourdieu (1984), where scholars organize around disciplines and scientific organizations, competing for the symbolic value of their activity. The concept of the field enables us to relate the objective structure of relations between scholars’ positions to a subjective space of position-takings, such as different epistemologies, mindsets, and values attributed to scientific methods, questions, and results. In Bourdieu’s work, the notion of habitus (Bourdieu 1979) conceptualizes the relationship between these two spaces: ‘Like the positions of which they are the product, habitus are differentiated; but they are also differentiating. Distinctive, distinguished, they are also operators of distinctions: they implement different principles of differentiation or use differently the common principles of differentiation’ (Bourdieu 1994: 23). Elias (2017: 75) emphasizes the importance of examining the organization and actual status of disciplines, such as biology, to understand the rivalries and solidarities that characterize the relations between institutions and the way they strive for relative autonomy. This programmatic view aligns with Bourdieu's invitation to compare different cases, with the hypothesis that the same essential power structure exists over the instruments of knowledge production, even if expressed in different ways and proportions (Bourdieu and Wacquant 1992).

### Strategy, Data, and Method

We draw our data from the Swiss Elite Database, which includes all full and associate professors across ten Swiss universities and two Federal institutes of technology. Thanks to the *Almanach des universités et hautes écoles suisses*, we were able to identify professors by university and faculty, with their last name, first name, date of birth, position held, and teaching chair. Following a prosopographical approach (Stone 1971; Rossier 2019), we collected extensive biographical and career data. Our analysis is focused on all 442 biology professors in Switzerland during 1957, 1980, and 2000. We identified professors' epistemologies and disciplines through an in-depth search of their institutional affiliations, research interests, and methods declared in their curriculum and publications. Additionally, we utilized Stettler's (2002) comprehensive collection of information on Swiss professors between 1945 and 1975, which also distinguish between disciplines and epistemologies. The association between the two is not straightforward and likely to change over time (Abbott 2001). Therefore, instead of using automatic classifications, we carefully classified each professor manually.

Some disciplines are strongly associated with specific epistemologies: molecular biology, biochemistry and field of microbiology are associated with functional epistemology, while ecology is associated to evolutionary biology. Some other disciplines are split into functional and evolutionary epistemologies. In 1957, zoology and botany were still mainly composed of traditional biologists, even if plant physiologists, for example, are included as functional biologists. To identify epistemologies, we refer to the full title of the professorship and, when it didn't allow us to identify epistemology, the declared research interests. For example, ‘zoology with a molecular focus’ was associated with functional biology. Anatomy and systematic biology were coded as evolutionary biology. Genetics, as a last example, also depends on the context and its classification relied on in-depth search on the declared research interests and publications of the professors.

Our study comprises three main steps. *First*, we examine the distribution of three types of resources – scientific, extra-scientific, and international – among Swiss biology professors in 1957, 1980, and 2000. By doing so, we aim to uncover patterns of inequality in the distribution of academic power. *Second*, we explore whether disciplinary and epistemological distinctions explain the observed similarities and differences between professors. We use the MCA to identify the resources that play a distinctive role in shaping the hierarchy of professors’ positions and disciplines. In the *third* step, we focus on the changing endowment in resources among evolutionary and functional biologists over time, and we analyze their socio-demographic characteristics. Here, we use the MCA to assess the position of each epistemology in relation to one another, and to examine the overrepresentation of specific modalities in each group of biologists. For each modality, we calculate a *p-value* and a *v-test*, which indicate the probability that the distribution in the classes is not due to chance (Husson et al. 2010). Table 1 shows all the modalities of active and supplementary variables used in the MCA.

**Table 1:** Sample and variables

Dimension	Modality	Cohort 1957	Cohort 1980	Cohort 2000	Total
Scientific resources	Number of cases	45	176	221	442
	Prestigious science award				
	Yes	9	36	37	82
	Type of tenure				
	Associate professorship	16	63	91	170
	Full professorship	29	113	130	272
	Postdoctoral stay				
	Elite institution	1	44	52	97
	Non-elite institution	18	63	104	185
	No	26	69	65	160
	Specialized research center				
	Yes	4	35	71	110
Extra-scientific resources	Member in SCNAT				
	Yes	4	11	12	27
	Member in SNSF				
	Yes	6	24	15	45
	Rector or dean				
	Yes	27	36	35	98
	Member in expert committee				
	Yes	12	24	13	49
	Extra-academic position				
	In-house lab	2	20	17	39
	Other	14	34	13	61
	No	29	122	191	342
International resources	Country of PhD				
	Switzerland	36	144	137	317
	Neighboring countries	8	21	43	72
	US	0	5*	25	30
	Other	1	6*	16	23
	Duration of pre-tenure phase abroad				
	1–4 years	7	66	104	177
	5–9 years	7	31	48	86
	10 years and more	3	17	26	46
	No	28	62*	43	133
Affiliations	Institutional affiliation				
	Biochemistry	2	46	58	106
	Botany	17	35	33	85
	Microbiology	8	36	58	102
	Molecular Biology	0	17	33	50
	Zoology	18	42	39	99
	Epistemology				
	Evolutionary	19	43	44	106
	Functional	26	133	177	336
Socio-demographic	Nationality				
	Swiss	35	139	133	307
	English-speaking countries	0	6	22	28
	French-speaking countries	3	7	9	19
	German-speaking countries	6	14	33	53
	Other coutries	1	7	13	21
	Missing	0	3	11	14
	Gender				
	Male	44	170	205	419
	Female	1	6	16	23
	Age of tenure in Switzerland				
	<=35 years	14	33	33	80
	36–40 years	12	68	85	165
	41–45 years	7	47	65	119
	46+ years	12	28	38	78

Note: * are displayed as *passive* in the MCA. The country of PhD: US and other respectively count for 2.8% and 3.4% of the sample for 1980, which are below the minimum of 5% per modality that is usually admitted in order not to over-estimate the relative weight of these modalities. Duration of pre-tenure phase: no is also set as *passive*, because it is redundant with the modality postdoctoral stay: no. Further details are displayed in the following section.

The *active* variables of the MCA are the following:

*Scientific resources* include:

- *Major scientific award* indicates the rewarding of at least one major prize, including the Nobel Prize and several prizes granted at the national level (Marcel Benoist Prize for biosciences, Otto Nägeli Prize for medical research, the Cloetta Prize for medical sciences and the Fredrich Miescher Prize for biosciences.
- *Type of tenure* discriminates between full and associate professorships.
- *Postdoctoral stay* in prestigious institutions refer to any academic positions held within a period of six years after the PhD obtention in one top ranked institution according to the Top 10 Shanghai Ranking universities for life sciences (2003), Shanghai’s Global Ranking of Academic Subjects for natural sciences (2017) and the World University Ranking 2018 for the life sciences.
- *Occupation in specialized research centers* indicates the occupation of a position in research institutions, which are anchored in national academic landscapes or funded by public-private partnerships or foundations. These include for example the Centre national de la recherche scientifique (CERN) in France, the Max-Planck Institute in Germany, the European Molecular Biology in Heidelberg (EMBL), the Scripps Clinic and Research Foundation in La Jolla, California, or the Friedrich Miescher Institute in Basel (FMI), Switzerland.

*Extra-scientific resources* include:

- *Member in SCNAT* indicates membership in the Central committee of the Swiss Academy of Natural Sciences, which is the main organization promoting the biological sciences in Switzerland.
- *Member in SNSF* indicates membership in the Council of the Swiss National Science Foundation, which is the main institution for research funding in Switzerland.
- *Rector or dean* informs about professors who served as rector of a Swiss university or dean of faculty, which are the two main executive functions in the Swiss higher education system.
- *Member in expert committee* informs about professors who served as experts in extra-parliamentary committees, who are engaged to respond to public administration or political issues.
- *Extra-academic position* refers to the occupation in a private company laboratory (in-house lab), or to any positions occupies in a non-academic organization, i.e., museums, botanical garden, or regional services.

*International resources* include:

- *Country of PhD* is indicating where the PhD was obtained: in Switzerland, neighboring countries (France, Germany, Italy), in the US, and all other countries.
- *Duration of the pre-tenured phase abroad* indicates the number of years of the career abroad before the tenure as professor in Switzerland.

The supplementary variables of the MCA are the following:

- *Institutional affiliation* refers to the discipline of teaching as from the *Almanach des universités des hautes écoles suisses* (biochemistry, botany, microbiology, molecular biology, and zoology).
- *Epistemology* distinguishes between evolutionary and functional biologists.

In addition, we consider the following socio-demographic variables: nationality, gender, and age of tenure in Switzerland.

### The Power Structure of Biological Sciences

To understand how scientific, extra-scientific, and international resources are distributed amongst professors, we conducted a specific MCA^1^ on 11 variables and 25 active modalities (7 dimensions, n=176 professors in 1980). We will later add 45 professors of 1957 and 221 professors of 2000 as supplementary individuals. The importance of each dimension for the interpretation are expressed by Benzecri’s modified rates (Le Roux and Rouanet 2010). Table 2 displays the variance and these modified rates for the first three axes of the MCA.

**Table 2:** Variance and modified rates

Dimension	1	2	3
Eigenvalue	0.18	0.16	0.12
Variance	13.4	12.3	9.3
Benzecri's modified rates (%)	46.4	33.7	8.4
Benzecri's modified rates cumulated (%)	46.4	80.1	88.5

**Fig. 1:**
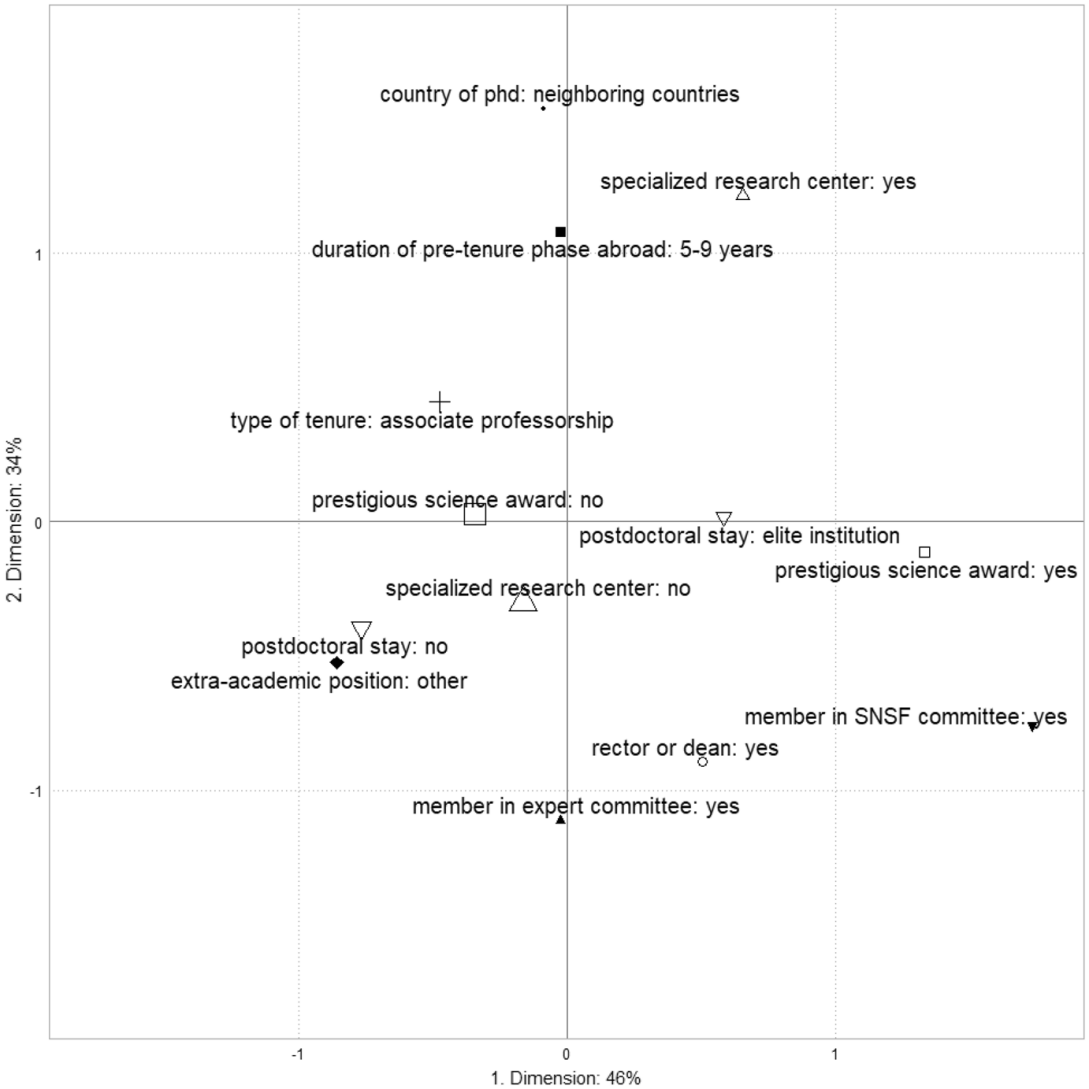
Distribution of the contributive modalities in the space of 1980 (axes 1 and 2)

The first axis has a modified rate of 46.4%, the second has a rate of 33.7%, and the third of 8.4%. We retained the two first axes for the interpretation of the results of the MCA, since the cumulative percentage of their modified rate is beyond 80% (Le Roux and Rouanet 2010). Figure 1 displays all contributive modalities which thus form the factorial plan of axes 1 and 2.

To interpret the structure of the factorial plan produced by the MCA, we examined the modalities that contribute above the average (4.0%) to the formation of the dimensions. Appendix 1 displays all the modalities that met this criterion. On the first axis, which has a Benzecri's modified rate of 46.4%, two distinct groups of professors emerge. On the left are those with a primarily local and extra-academic focus, as indicated by their lack of experience with postdoctoral stays outside Switzerland (11.9%), their ability to leverage non-academic experience (7.3%) to achieve associate professorships (4.2%), and their lack of major scientific prizes (4.8%). On the right are professors with high levels of symbolic credibility, including membership in the committee of the SNSF (21.1%), major scientific prizes (18.7%), positions in specialized research centers (4.4%), and postdoctoral stays at elite institutions (4.4%). In other words, the hierarchy among professors in 1980 is primarily structured by an opposition between those with weaker scientific resources and those with a high degree of symbolic recognition.

The second axis, with a modified rate of 33.7%, reflects the opposition between international and institutional capitals. The top of the figure represents professors who have held positions in specialized research centers (16.5%), obtained their PhD degrees in neighboring countries (15.9%), experienced a pre-tenure phase abroad for 5 to 9 years (11.5%), and achieved associate professorship (4.0%). On the bottom of the figure, we find professors who are members of expert committees (9.5%), rectors or deans (9.2%), members of the SNSF (4.4%), and who have never held positions in specialized research centers (4.1%). Therefore, the hierarchy within the space of professors in 1980 is secondarily structured on the opposition between international and institutional capitals.

**Fig. 2:**
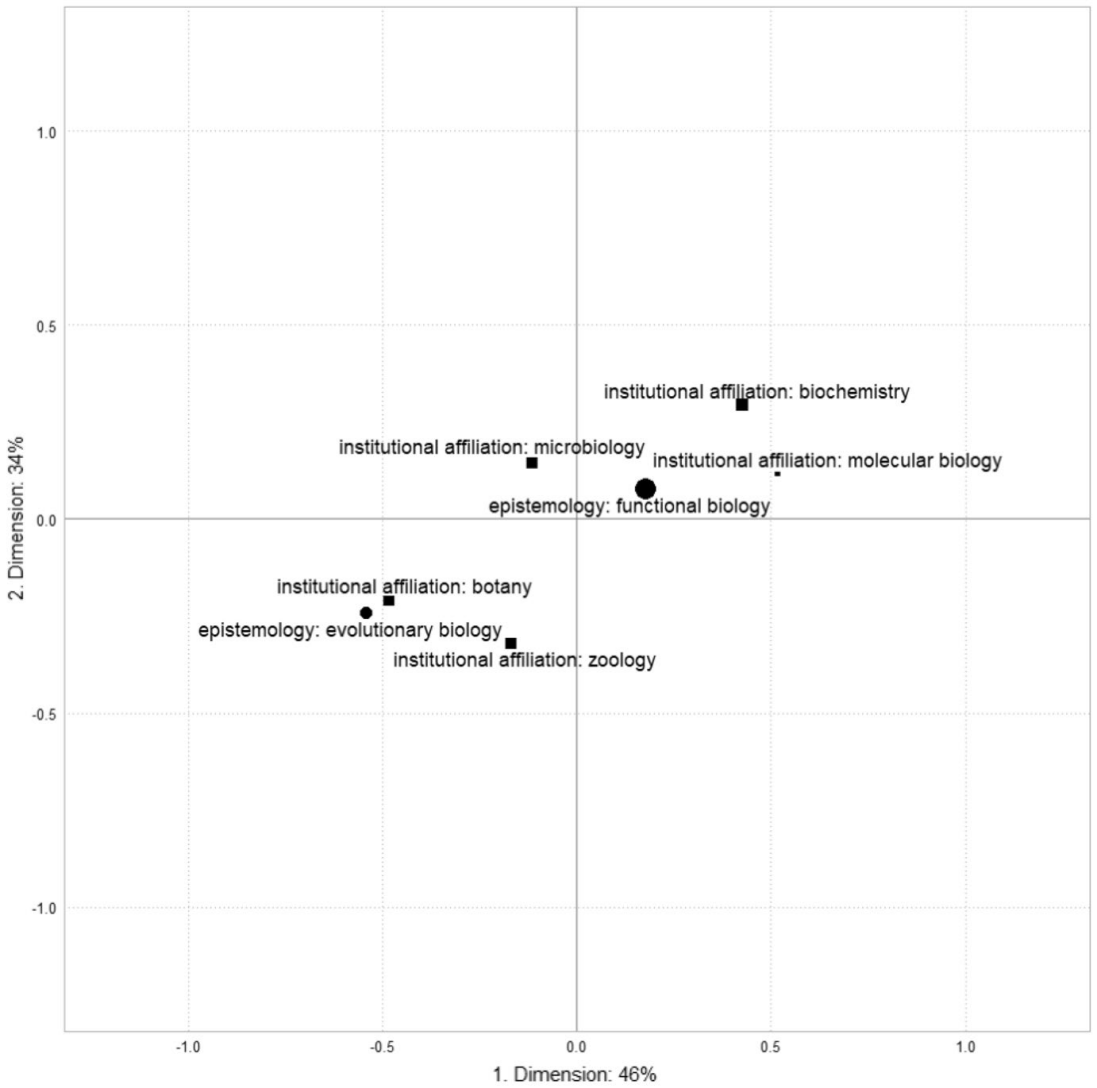
Disciplinary affiliations and epistemologies (1980)

The way disciplinary affiliations are projected into the space of objective relations between professors’ positions informs us about the relationship between the structure of power and the symbolic hierarchy of disciplines. The points in Figure 2 represent the barycenter of all representatives.

Figure 2 reveals that both epistemologies and disciplinary affiliations are primarily situated along the first dimension, which corresponds to the volume of scientific capital. To interpret the distances between the coordinates of the modalities, we follow the rule that a difference of 0.5 or more is *notable*, while a difference of 1 or more is *significant* (Le Roux and Rouanet 2010: 59; Hjellbrekke 2018: 64). Differences of less than 0.5 are not considered for interpretation. Evolutionary biologists stand out from functional biologists along the first axis, reflecting their higher level of scientific capital. However, the second axis, which represents the opposition between international and institutional capitals, does not appear to strongly differentiate between epistemologies. Epistemologies are also closely related to disciplines. For instance, molecular biologists are significantly different from botanists on the right-hand side of the figure. Notable differences also exist, with molecular biologists and biochemists having higher levels of scientific capital compared to microbiologists, zoologists, and botanists. The symbolic hierarchy of disciplines identified in the literature is clearly reflected in the display of modalities, particularly the high levels of symbolic credibility associated with biochemistry and molecular biology. However, microbiologists who do not belong to these fields do not appear to differ from zoologists and botanists in terms of scientific capital. To fully understand this structure, further investigation into its historical transformations is necessary.

### Time Variations of the Symbolic Hierarchy of Epistemologies

**Fig. 3:**
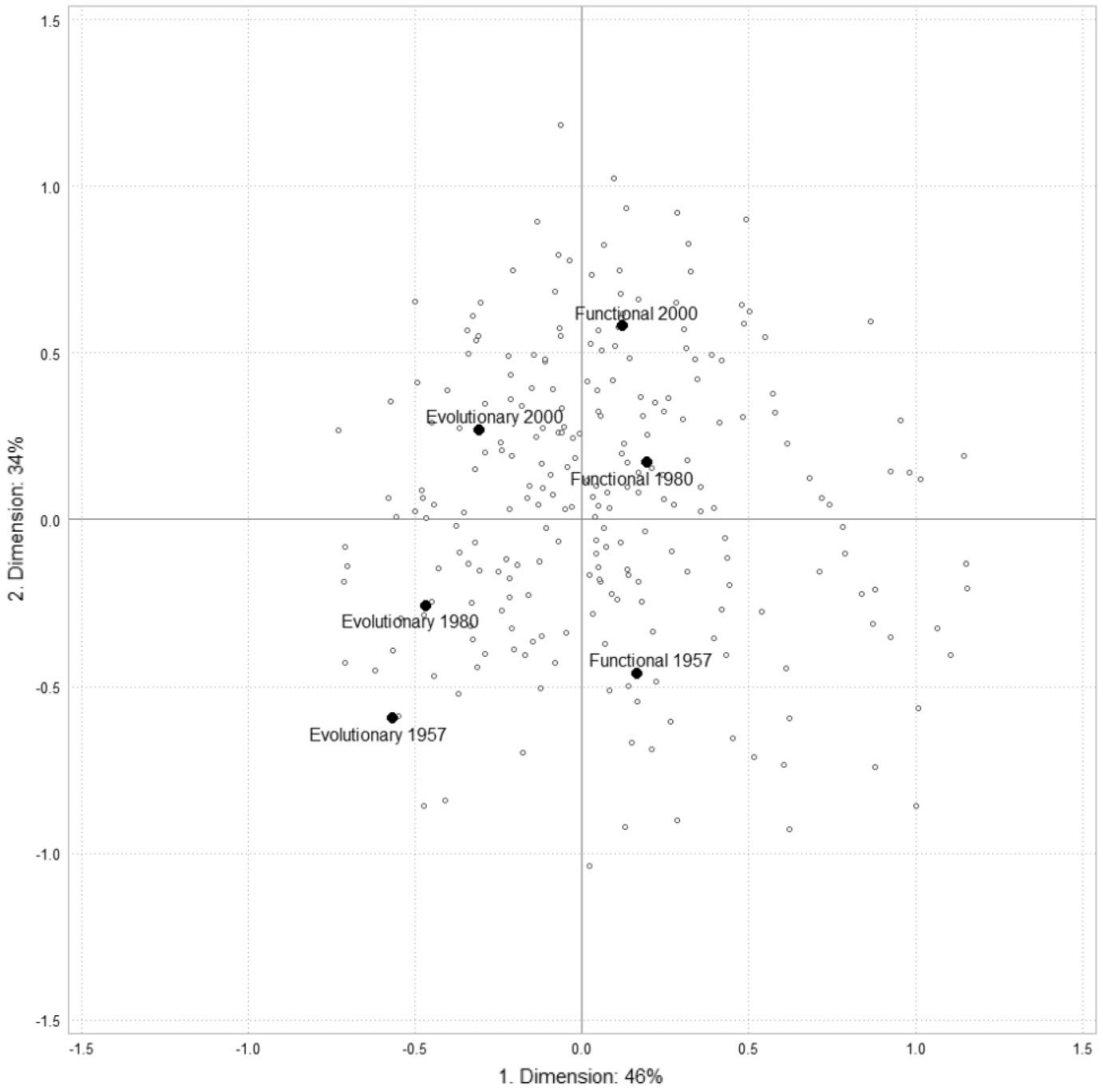
Epistemologies (cohorts)

To analyze the historical changes in the symbolic hierarchies of epistemologies, we included all professors in 1957 (n=45) and 2000 (n=221) as supplementary individuals projected onto the space. The positioning of these individuals was determined with reference to the structure of the space in 1980. By using the coordinates of these supplementary individuals, we were able to map the positions of disciplinary affiliations across the cohorts (as shown in Figure 3). This approach allowed us to explore how the symbolic hierarchy of disciplines and epistemologies have evolved over time and identify any significant changes in their positioning within the space.

The internationalization of the scientific field is a well-documented trend that is reflected in both evolutionary and functional epistemologies (Gingras 2002; Goastellec and Pekari 2013). Over time, both epistemologies have increasingly relied on international resources to advance their research agendas, which has contributed to their growing influence in the scientific community. However, while this trend has led to a notable decrease in the symbolic credibility of both epistemologies, it has not bridged the gap between them in terms of their overall scientific capital. In other words, the global internationalization of the biological sciences has not had a significant impact on the relative status of these two epistemologies.

This structural divide between evolutionary and functional biologists is reflected in their different profiles, as shown in Table 3. The table presents the percentages of various modalities that are overrepresented among each group of biologists, based on data from three different cohorts (1957, 1980, and 2000).

**Table 3:** Over-represented modalities

Cohort	Evolutionary			Functional			Mean
	1957	1980	2000	1957	1980	2000	
Cases (n)	19	43	44	26	133	177	442
Prestigious science award: yes	9.5	2	0	28.6	**26.5**	19	18.6
Postdoctoral stay: elite institution	0	15.7	11.8	3.6	**29.5**	24.6	21.9
Postdoctoral stay: no	**61.9**	**49**	44.1	**57.1**	28.9	30.3	36.2
Specialized research center : yes	4.8	5.9	8.8	10.7	25.9	**40.1**	24.9
Member in SNSF: yes	4.8	2	5.9	**25**	**15.7**	5.6	10.2
Rector or dean: yes	**57.1**	23.5	14.7	**64.3**	19.9	12.7	22.2
Member in expert committee: yes	**28.6**	**21.6**	5.9	**25**	10.2	4.2	11.1
Extra-academic position: in-house lab	4.8	0	2.9	3.6	**13.9**	9.2	8.8
Extra-academic position: other	**42.9**	**27.5**	17.6	21.4	13.3	2.8	13.8
Extra-academic position: no	52.4	72.5	79.4	75	72.9	**88**	77.4
Country of PhD: Switzerland	85.7	**84.3**	55.9	75	**80.7**	57.7	71.7
Country of PhD: neighboring countries	14.3	7.8	**29.4**	21.4	13.9	18.3	16.3
Country of PhD: US	0	0	5.9	0	3	**16.2**	6.8
Duration of pre-tenure phase abroad: 1-4 years	4.8	37.3	38.2	21.4	42.2	**47.9**	40
Duration of pre-tenure phase abroad: no	**90.5**	45.1	23.5	46.4	27.1	17.6	30.1
Nationality: Swiss	**90.5**	**84.3**	52.9	71.4	**76.5**	56.3	69.5
Nationality: English speaking countries	0	5.9	11.8	0	2.4	**12**	6.3
Nationality: German speaking countries	9.5	5.9	**29.4**	14.3	9.6	12.7	12
Nationality: other coutries	0	0	0	3.6	4.8	**8.5**	4.8
Age of tenure in Switzerland: <=35 years	33.3	23.5	17.6	**35.7**	19.9	8.5	18.1
Age of tenure in Switzerland: 36-40 years	23.8	37.3	26.5	25	**44.6**	35.9	37.3
Age of tenure in Switzerland: 41-45 years	9.5	15.7	**44.1**	17.9	26.5	31.7	26.9
Age of tenure in Switzerland: 46+ years	33.3	23.5	11.8	21.4	9	**23.9**	17.6

Note: modalities with a v-test equal or superior to 2 are displayed in **bold**.

In the academic field of biology, there exists a notable disparity between functional and evolutionary biologists in terms of their distribution of resources. Professors in functional biology tend to be well-endowed in both scientific and international capitals: they are more likely to pursue postdoctoral positions at elite institutions, resulting in prestigious scientific awards. Functional biologists gain scientific credibility through their positions in specialized research centers, particularly in the 2000 cohort. In contrast, professors in evolutionary biology are historically more locally grounded and engaged in extra-academic pursuits: they tend to remain in Switzerland and pursue non-academic careers. While both groups of biologists have held leadership positions in academic institutions, such as rector and dean, there is a general trend of disaffection among biologists towards such roles. However, it is noteworthy that evolutionary biologists have remained the reference persons for public authorities for a longer period of time.

One key aspect that distinguishes between functional and evolutionary biologists is their differentiated involvement in extra-academic activities. On the one hand, evolutionary biologists were regularly invited as experts by the political authorities from 1957 to 1980. They were also likely to pursue a professional career outside of academia, such as in public services. In 2000, these two forms of extra-academic activities were much less frequent, suggesting a trend towards academicization of the professors’ careers. On the other hand, professors in functional biology are prominent in in-house laboratories, which is reflective of the discipline's integration in the medical, pharmaceutical, and agri-food biotechnology sectors. However, this situation is characteristic of the 1980s and in 2000 the professors are very less likely to occupy such professional positions outside academia. It must be noticed that we do not refer to potential involvements as advisors in *startup* or other mandates that are not fully professional positions. Even though there are many links between (functional) biology and industry, at professor level, careers have become more academic. In other words, the value attributed to extra-academic careers has diminished as a condition for the tenure, echoing a global trend towards the generalization of the doctorate as a condition of access to academia, as well as the increasing formalization of scientific careers (Musselin 2005; Benz et al. 2021).

We also find variations in socio-demographic characteristics among evolutionary and functional biologists. The process of internationalization of the academic field has resulted in the increasing importance of different nationalities depending on the epistemological approach. In the case of evolutionary biologists, Swiss nationality is overrepresented, although German-speaking countries have become increasingly important since 2000. Conversely, English-speaking countries are overrepresented among functional biologists in the most recent period, which partly explains the over-representation of doctorates obtained in the US. Furthermore, the differentiated career paths of evolutionary and functional biologists lead to a generally higher age of appointment, reflecting the trend of longer postdoctoral periods and fewer tenured appointments in academia (Fumasoli and Goastellec 2015; Sarrico 2022).

## Discussion

Our study reveals that the evolutionary and functional epistemologies within the biological sciences have remained distinct from each other over time, with no apparent convergence towards a shared approach. This lack of reconciliation can be attributed to the persistent structural conditions that shape the symbolic hierarchy of disciplines and resources within the field. Our analysis of professors' resource endowments across three cohorts (1957, 1980, and 2000) shows that the prestige of different biological disciplines is closely linked to the volume of scientific resources they command. Specifically, functional biologists tend to concentrate resources and prestige, while evolutionary biologists prioritize local roots and extra-academic careers. Although there have been some changes in the symbolic valuation of scientific and institutional resources in the 2000 cohort, these changes have not yet significantly affected the divide between epistemologies. Our findings suggest that the division between evolutionary and functional biologists remains entrenched in the relational structure of symbolic credibility. Any efforts towards a reconciliation between these approaches must take into account the specific resources and positions of scholars who seek to bridge the divide. It is also important to acknowledge the lasting impact of the structural distinction between these two epistemologies, which continues to shape the field of biology even today.

The sociological issue at hand pertains to the relationship between the structure of academic power and the range of possibilities available for different epistemologies to coexist (Bourdieu 1992). To examine this, we have delved into the unequal distribution of resources amongst professors who hold the power to define scientific practices and shape the mindset of their peers. The scientific background of researchers plays a critical role in shaping their practices and influencing their perceptions about what constitutes legitimate scientific inquiry and how it should be conducted. It is therefore evident that epistemologies and the disciplines that they are based upon are inherently tied to academic power structures. Through our analysis, we have established that the symbolic hierarchy of disciplines is shaped by the volume and type of scientific resources at their disposal.

While the diminishing symbolic distinction between functional and evolutionary epistemologies may appear to be conducive to a reconciliation, it may also be argued that the trend could in fact limit the possibility of a dialogue. One reason for this is that while evolutionary biologists have gained international resources, they have lost a crucial source of counter-power as they have retreated from institutional and extra-academic positions. In contrast, functional biology has been increasingly labelled as rational by political authorities and funding agencies, and as a result, has acquired the status of big science (Strasser 2006; Rheinberger 2010; Morange 2020). This has benefited not only molecular biologists, but also biochemists and microbiologists, thereby reinforcing the autonomy of functional biology. Consequently, the chances of evolutionary biologists being recruited on extra-academic criteria have decreased. Furthermore, professional experience in non-academic institutions is no longer as valued, and professors who hold the most symbolic credit at the end of the 20th century have followed very academic careers. This is not to say that externalities outside the academy have disappeared – startups and patents are now an essential part of the scientific world – but it does mean that these links are not forged by occupying professional positions outside academia. The tightening of the symbolic hierarchy of disciplines on scientific criteria is not only related to the symbolic status of functional biology but is also reinforced by the growing autonomy of evolutionary biologists who are now in direct competition with functional biologists. While evolutionary biologists were previously recruited for their expertise in public policy making, this alternative source of legitimacy has declined in favor of scientific resources. Overall, this suggests that the changing structure of academic power has important implications for the relationship between functional and evolutionary epistemologies.

Mayr's (1961) division between evolutionary and functional biologists extends beyond a mere epistemological debate and shapes the entire structure of scientific careers and academic power dynamics. While there have been efforts to promote the integration of epistemologies, these initiatives are viewed with skepticism due to the entrenched power dynamics within the field. This structural inequality presents a significant challenge to productive dialogue on large-scale environmental changes and their impact. Despite science being regarded as a shared framework for addressing these challenges (Saint-Martin 2020), an excessive focus on scientific criteria can hinder interdisciplinary collaboration. These observations underline the institutional constraints that limit the possibility of developing a reconciled scientific approach to address current poly-crisis issues. Functional epistemology remains deeply ingrained in the contemporary biological sciences and is held by those who occupy dominant positions in the field. Therefore, the efforts to reconcile epistemologies through the discussion of specific notions and concepts (Rheinberger 2010; Morange 2011) are insufficient to overcome disciplinary and academic power structures that serve as central mechanisms for knowledge regulation and circulation (Méthot 2022).

## Conclusion

In light of global crises such as climate change and the Covid-19 pandemic, the biological sciences have been increasingly called upon to participate in public debates and policy campaigns. As a result, previously marginalized evolutionary epistemologies have gained traction, and calls for a reconciliation with functional epistemologies have emerged within the biological sciences. This article aimed to shed light on the mechanisms of academic power that underlie the symbolic hierarchy of the biological sciences and how differences between functional and evolutionary biology have evolved over time. To address these questions, we conducted a study using data on all tenured professors of biology in Switzerland in 1957, 1980, and 2000, assessing their access to scientific, extra-scientific, and international resources. Our findings highlight how institutional affiliations and both evolutionary and functional epistemologies are shaped by academic power relations and correspond to specific professors’ access to resources.

Drawing on Bourdieu's theory of practice, we emphasize the embeddedness of practices and mindsets in social relations. The divide between evolutionary and functional biologists not only differentiates careers and resources but also engages in a disagreement about the definition of life. This divide poses a significant obstacle to fruitful perspectives of reconciliation. Furthermore, the recent tendency to define symbolic credibility based on scientific criteria is disadvantageous to evolutionary biology, which has limited access to such resources. While this divide is historically rooted, historical transformations of the academic field demonstrate that changes at the margin can have structural effects later on. The literature on molecular biology shows that political and economic powers can influence the symbolism of disciplines (Magner 2002; Morange 2020). The recent initiatives of political ecology and the efforts of both scientists and citizens to recognize the climatic and environmental stakes could benefit future attempts to rehabilitate evolutionary biology and its potential for dialogue with other scientific disciplines if the criteria of the hierarchy of knowledge are reconsidered.

The increasing interest in the relationship between biological and social sciences (Quilley and Loyal 2005; Meloni et al. 2016; Laland 2017) offers opportunities to explore the political dimensions of epistemologies (Louvel 2020). While some resistance persists (Heilbron and Gingras 2015), more research is needed to investigate the role of academic power in shaping epistemological divides. Future studies may benefit from focusing on the margins of structural dynamics, where change may occur more rapidly, to deepen our understanding of the relationship between epistemologies and power. We suggest that exploring these connections could have important implications for understanding and addressing the complex challenges facing society today, including environmental crises and the need for interdisciplinary collaboration.

## Appendix 1. Contributions of modalities to axes 1 and 2

**Table:**

Variable	Modality	Ctr	Coord
Dimension 1: right-side (+)			
Member in SNSF	Yes	21.1	1.73
Prestigious science award	Yes	18.7	1.33
Postdoctoral stay	Elite institution	4.4	0.59
Specialized research center	Yes	4.4	0.66
Dimension 1: left-side (−)			
Postdoctoral stay	No	11.9	− 0.76
Extra-academic position	Other	7.3	− 0.86
Prestigious science award	No	4.8	− 0.34
Type of tenure	Associate professorship	4.2	− 0.47
Dimension 2: top (+)			
Specialized research center	Yes	16.5	1.21
Country of PhD	Neighboring countries	15.9	1.54
Duration of pre-tenure phase abroad	5-9 years	11.5	1.08
Type of tenure	Associate professorship	4.0	0.45
Dimension 2: bottom (−)			
Member in expert committee	Yes	9.5	− 1.11
Rector or dean	Yes	9.2	− 0.89
Member in SNSF	Yes	4.4	− 0.76
Specialized research center	No	4.1	− 0.30

## Footnotes

1 Specific MCA allows us to consider certain modalities as passive by making them non-contributory to the variance. This MCA was produced using the *soc.ca* package for R software (Larsen et al. 2021).
